# Tuberculosis among refugees and migrant populations: Systematic review

**DOI:** 10.1371/journal.pone.0268696

**Published:** 2022-06-09

**Authors:** Abyot Meaza, Habteyes Hailu Tola, Kirubel Eshetu, Tedla Mindaye, Girmay Medhin, Balako Gumi

**Affiliations:** 1 Ethiopian Public Health Institute (EPHI), Addis Ababa, Ethiopia; 2 Aklilu Lemma Institute of Pathobiology, Addis Ababa University, Addis Ababa, Ethiopia; 3 Sibley Memorial Hospital, Johns Hopkins Medicine, Washington, DC, United States of America; Gulu University, UGANDA

## Abstract

Tuberculosis (TB) is an important cause of morbidity and mortality among refugees and migrant populations. These groups are among the most vulnerable populations at increased risk of developing TB. However, there is no systematic review that attempts to summarize TB among refugees and migrant populations. This study aimed to summarize evidence on the magnitude of TB among refugees and migrant populations. The findings of this review will provide evidence to improve TB prevention and control policies in refugees and migrants in refugee camps and in migrant-hosting countries. A systematic search was done to retrieve the articles published from 2014 to 2021 in English language from electronic databases. Key searching terms were used in both free text and Medical Subject Heading (MeSH). Articles which had reported the magnitude of TB among refugees and migrant populations were included in the review. We assessed the risk of bias, and quality of the included studies with a modified version of the Newcastle–Ottawa Scale (NOS). Included studies which had reported incidence or prevalence data were eligible for data synthesis. The results were shown as summary tables. In the present review, more than 3 million refugees and migrants were screened for TB with the data collection period between 1991 and 2017 among the included studies. The incidence and prevalence of TB ranged from 19 to 754 cases per 100,000 population and 18.7 to 535 cases per 100,000 population respectively among the included studies. The current findings show that the most reported countries of origin in TB cases among refugees and migrants were from Asia and Africa; and the incidence and prevalence of TB among refugees and migrant populations is higher than in the host countries. This implies the need to implement and improve TB prevention and control in refugees and migrant populations globally.

**Trial registration**: The protocol of this review was registered on PROSPERO (International prospective register of systematic reviews) with ID number, CRD42020157619.

## Introduction

Tuberculosis (TB) is an infectious disease caused by the bacillus *Mycobacterium tuberculosis* [1]. It typically affects the lungs (pulmonary TB) but can also affect other sites (extra pulmonary TB) [2]. TB is causing morbidity and mortality each year among millions across the world. It is the tenth leading cause of death worldwide and the leading cause of a single infectious agent [3]. The recent World Health Organization (WHO) recent report indicated 10 million new cases and 1.4 million deaths occurred globally [3].

Tuberculosis is an important cause of morbidity and mortality among refugees and migrant populations [4]. Refugees are defined as people who are outside their country and cannot return due to a well-founded fear of persecution because of their race, religion, nationality, political opinion or membership in a particular social group [5]. Whereas, migrants are defined as people and/or family members moving to another country or region to better their material or social conditions and improve the prospects for themselves or their family [6].

The number of international migrants reached 244 million in 2015, with almost two-thirds travelling to industrialized countries [7]. The number of refugees also exceeded 70 million in 2018, which was the highest level that the United Nation High Commission for Refugees (UNHCR) has seen in its almost 70 years [8]. Sub-Saharan Africa hosted more than 26% of the world’s refugee population, and the number of refugees in the Horn of Africa exceeded 3 million, with Uganda (1.1 million) and Ethiopia (905,000) hosting the largest numbers [9].

Refugees and migrants are the most affected populations at risk of developing TB and other infectious diseases due to their living conditions [7]. Poor living conditions and overcrowding in refugee settlements potentially increase the risk of TB infection [10]. The impact of migration on TB epidemiology is high [11]. The arrival of large groups of refugees can affect TB control efforts in receiving countries by significantly increasing the disease burden and cost of health services [10]. Since the majority of the migrants originate from low-income countries with a high TB burden, it imposes new challenges for global TB control and eradication towards achieving the stop TB strategy [12].

Although there is published evidence and reports in different geographical areas [4, 13–18], there is a lack of systematic review that attempts to summarize the magnitude of TB among refugees and migrant populations. The present systematic review aimed to summarize existing published literature on the magnitude (incidence and prevalence) of TB among refugees and migrant populations living in refugee camps and in migrant-hosting countries.

## Methods

### Search strategy

We conducted a systematic review of published articles and summarized findings about the incidence and prevalence of TB among refugees and migrant populations living in refugee camps and in migrant-hosting countries following PRISMA (Preferred Reporting Items for Systematic Reviews and Meta-Analyses) standards [19] (S1 File).

We systematically searched electronic databases: Medline/PubMed, Web of Science and Google scholar for English language articles. We used sensitive searching method to include all potential articles in the data bases. The search was restricted to papers published in the last 7 years (between 2014 and 2021). The electronic database search was conducted from 01 August 2021 to 4 November 2021. We used a search strategy by combining key-terms: "Tuberculosis", "Multidrug-Resistant", "Pulmonary Tuberculosis", "Pleural Tuberculosis", "Miliary Tuberculosis", "Meningeal Tuberculosis", "Lymph node Tuberculosis", "Laryngeal Tuberculosis", "Mycobacterium tuberculosis", "Prevalence", "Epidemiology", "Cross-Sectional Studies", "Incidence", "Cohort Studies", "mortality", "Refugees", "Refugee Camps", "Migrant" and "Transients and Migrants" both in MeSH and free text terms.

### Inclusion and exclusion criteria

We included cross-sectional studies, retrospective cohort studies and cohort studies which had reported incidence or prevalence as an outcome variable. Eligible studies which were conducted in different parts of the world and published from 2014 to 2021 were included in the review. We excluded studies with different outcome variables, and studies published in non-English languages. The included studies were grouped as incidence and prevalence for the syntheses.

### Study selection process

**Fig 1:**
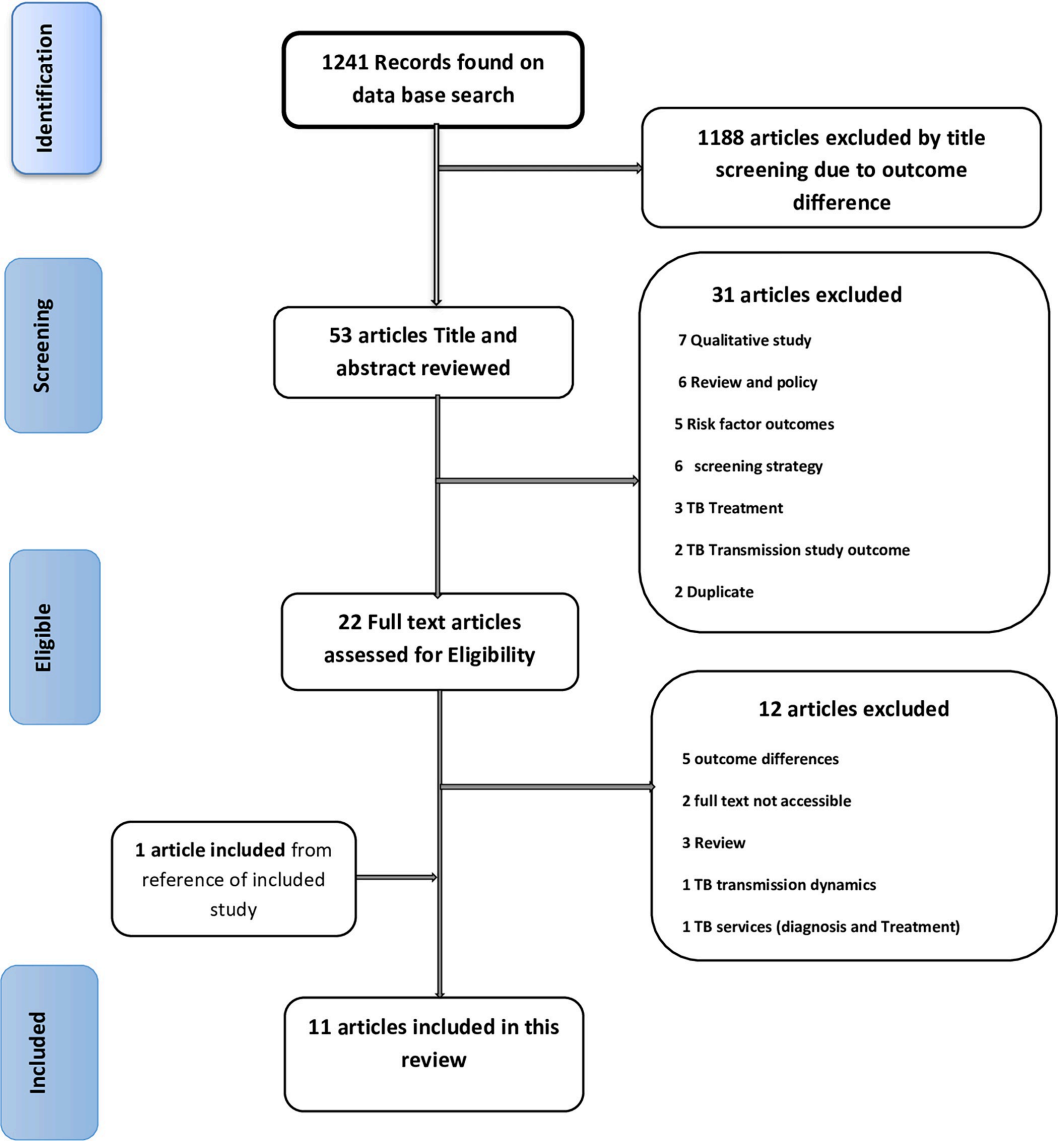
Selection process flow diagram.

A total of 1241 records were identified from data base searches and 1188 articles were excluded after title screening due to outcome variable difference with the present review objective. Upon title and abstract screening of 53 articles, 31 articles were excluded based on the exclusion criteria. Then the remaining 22 articles in full text were reviewed for eligibility and 12 articles were excluded. In the final grade, 10 articles remained to be included in the review and one article was also selected from the bibliography of the included study. Thus, a total of 11 studies were included in this systematic review (Fig 1). Eligibility assessment was performed independently in an unblinded standardized manner and disagreements were resolved by discussion.

### Study quality assessment

We assessed the risk of bias, certainty and quality of the included studies with a modified version of the Newcastle–Ottawa Scale [20]. The scale assesses three key points of a given study: participant selection; comparability of the groups; and outcome (s) assessment. We assigned stars for each point of the scale to categorize the studies into good, fair and poor quality based on the NOS [20]. A good quality study was scored 3 or 4 stars in participant selection, 1 or 2 stars in comparability of groups, and 2 or 3 stars in outcome (s) assessment. A fair quality study was scored 2 stars in participant selection, 1 or 2 stars in comparability of the groups, and 2 or 3 stars in outcome (s) assessment. A poor-quality study was scored 0 or 1 star in participant selection, 0 stars in comparability of the groups and 0 or 1 star in outcome (s) assessment (S2 File). In the case of disparity between the two authors (AM and HH) during the study selection process, the disparities were resolved by the decision of the second author (KE).

### Data extraction

We developed a data extraction format based on the Cochrane Consumers and Communication Review Group’s data extraction template [21]. The template was pilot-tested on 3 randomly-selected included studies, and refined accordingly. One review author (AM) extracted the data from included studies and the second author (HH) checked the extracted data. Disagreements were resolved by discussion between the two authors and a third author (KE). We extracted information on characteristics of participants such as age range, mean of age, sex ratio and county of origin of refugees and migrant populations. Study characteristics such as first author and year of publication; study location; study population; design; sample size; year (s) data were collected; TB diagnosis method; reported burden of TB (incidence, and prevalence per 100 000 population).

### Ethics consideration

Ethical approval was not required, as this review was based on previously published articles. However, the protocol of this review was registered on PROSPERO, University of York, Centre for Reviews and Dissemination and can be accessed with ID number CRD42020157619 (S3 File).

### Statistical analysis

Extracted data were summarized using Microsoft excel version 16 and exported to STATA version 14 for statistical analysis. Age range, mean age, sex ratio and the most reported countries of origin in TB cases among refugees and migrants were summarized to describe study characteristics. The reported burden of TB (incidence and prevalence) was summarized in a table. TB incidence and/or prevalence were calculated if the number of TB cases and population estimates were provided. Included studies which had reported the above data were eligible for data synthesis. Risk of bias due to missing results were minimized by inclusion of results from sources.

## Results

**Fig 2:**
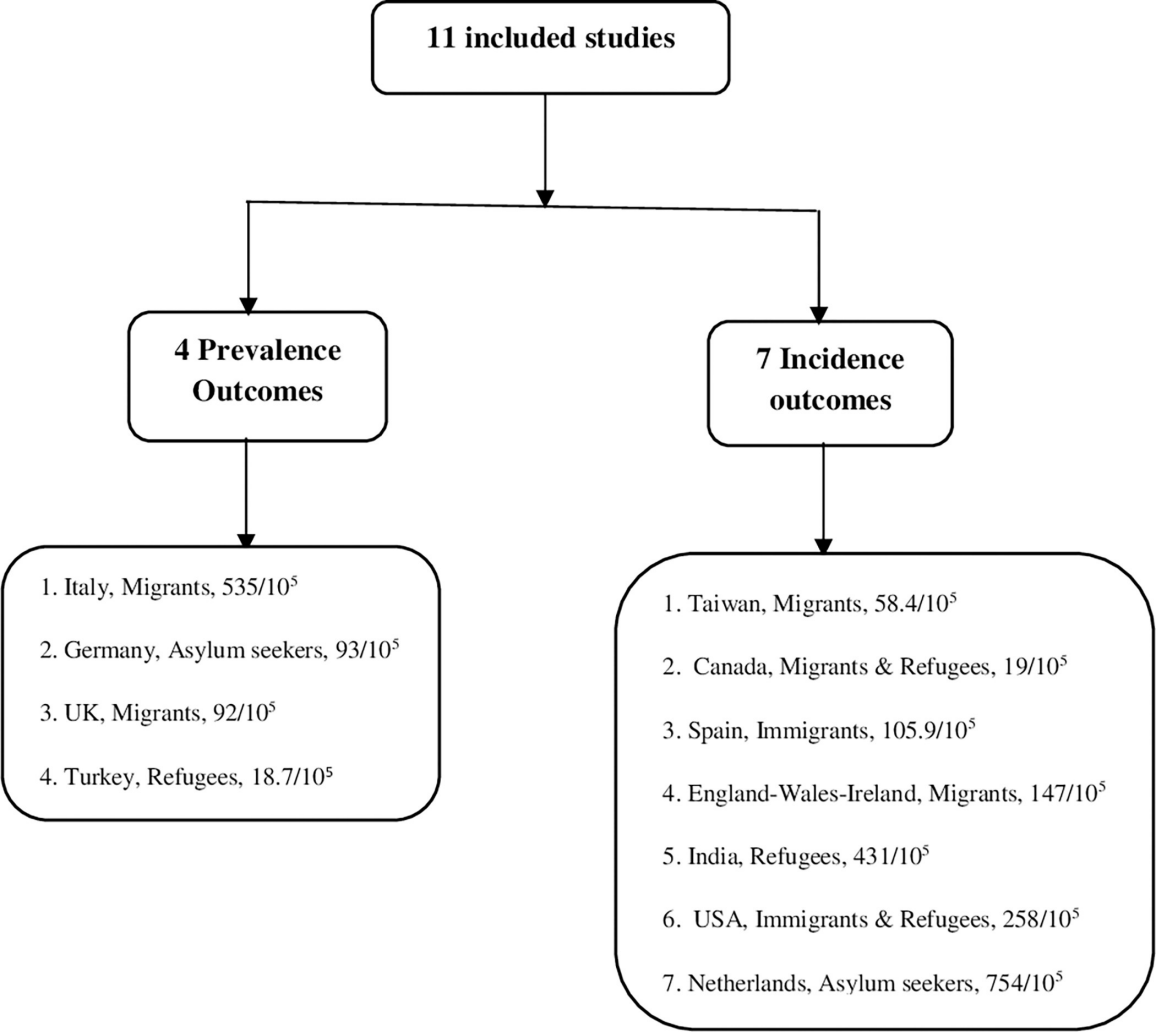
Results of 11 included studies.

Elven articles published from studies conducted in 10 countries and addressed TB in migrants, refugees, and asylum seekers. Nine articles were published on refugees and migrants in Europe, three studies in Asia, one study in the United States of America and one in Canada. Half of the included articles (6/11) were published in 2016. The country of origin of refugees and migrants screened for TB varied widely across regions and countries (Fig 2).

Two studies confirmed TB with chest X-Ray (CXR), and three studies confirmed TB diagnosis with bacteriological examination. Both CXR and bacteriological examination used by three studies; and clinical, CXR & bacteriological examination used by two studies as the method of TB diagnosis (Tables 1 and 2). The design and scope of the studies varied across the included studies. Six cross sectional studies, three retrospective cohort studies, one cohort study and one study with a scientific report were included in the present review (Tables 1 and 2). We observed heterogeneity among results of the included studies by grouping the study results in to incidence and prevalence.

**Table 1:** Study characteristics and TB Incidence results among the included studies.

First author, year of publication	Study location	Design	Study population	Sample size	Year(s) data collected	Type of TB diagnosis	Incidence
Lu et al., 2019 [15]	Taiwan	Retrospective Cohort	Migrants	379422	2004 to 2013	CXR	58.4 per 10^5^
							*Taiwan*: *45*.*5–76*.*8 per 10*^*5*^
Asadi et al., 2017 [22]	Alberta, Canada	Retrospective cohort	Migrants and refugees	223225	2002 to 2013	Culture	19/10^5^
							Alberta: 4.7/10^5^
Ospinia et al., 2016 [23]	Spain	Cross sectional	Immigrants	3284	1991 to 2013	CXR and Bacteriologic	105.9/10^5^
							Highest Pakistan, India, Bangladesh (675/10^5^), followed by Africa (329/10^5^)
Aldridge et al., 2016 [16]	UK	Cohort	Migrants	519955	2006 to 2012	CXR and Bacteriologic	147/10^5^
Dierberg et al., 2016 [24]	India	Cross sectional	Refugees	27714	September 2011 to March 2013	CXR, Bacteriologic and Xpert MTB/RIF	431 cases/10^5^
							*India*: *181 cases/10*^*5*^
Liu et al., 2016 [25]	USA	Cross sectional	Immigrants and refugees	1561460	2007 to 2012	Culture based examination	258 cases per 10^5^
Boogaard et al, 2020 [26]	Netherlands	retrospective cohort	Asylum seekers	26,057	January, 2013, to December 2017	Clinical, CXR, Bacteriologic	754 cases per 10^5^
							*Netherlands*: *4*.*6/10*^*5*^

**Table 2:** Study characteristics and TB prevalence results among the included studies.

First author, year of publication	Study location	Design	Study population	Sample size	Year(s) data collected	Type of TB diagnosis	Prevalence
Vanino et al., 2017 [27]	Bologna, northern Italy	Cross sectional	Migrants	3366	2014 to 2015	CXR	535/10^5^
							*Italy*: *6*.*7/10*^*5*^
Meir et al., 2016 [28]	Friedland, Germany	Cross sectional	Asylum seekers	11773	b/n 2014 and2015	Clinical, CXR and Bacteriologic	93/10^5^
							*Germany*: *5*.*3/10*^*5*^
Aldridge et al., 2016 [29]	UK	Cross sectional	Migrants	476455	between 2005 and 2013	Bacteriologic confirmation	92 per 10^5^
Ismail et al, 2018 [30]	Turkey	Scientific report	Refugees	10689	ND	ND	18.7/10^5^

ND-Not Described.

More than 3 million refugees and migrants were screened for TB using clinical history of TB, CXR, and bacteriological examination mainly in hospital and reception settings. Ten studies collected data between 2002 and 2017, while one study collected data between 1991 and 2013 (Tables 1 and 2).

Eleven studies have reported the incidence or prevalence of TB in refugees and migrant populations. The incidence of TB ranged from 19 to 754 cases per 100,000 population among the seven included studies (Table 1) and the prevalence of TB ranged from 18.7 to 535 cases per 100,000 population among the four included studies (Table 2).

More than half of the studies had reported TB cases whose age ranges were between 13 to 54 years and males were higher in reported TB cases as compared to females. The most reported countries of origin in TB cases among refugees and migrants were from Asia and Africa. India, Philippines, Pakistan, Syria, Thailand and Vietnam were the most reported countries of origin in TB cases from Asia, whereas Somalia and Eretria were the most reported countries in TB cases from Africa (Table 3).

**Table 3:** Age range, gender and country of origin of notified TB cases across the included studies.

First author, year of publication	Age range of TB cases	Male: Female ratio of TB cases	Country of origin
Lu et al., 2019 [15]	45–54	Higher in males (3:1)	Indonesia, Philippines, Thailand, and Vietnam
Asadi et al., 2017 [22]	13–37	Higher in males	Philippines, India, or China
Vanino et al., 2017 [27]	18–41	All are males (18/18 males)	West Africa and India
Meir et al., 2016 [28]	Mean age 31.2Yrs	ND	Eritrea, Russia, Pakistan and Syria
Ospinia et al., 2016 [23]	25–44 years	Highest in Males (66.7%)	Latin America, Pakistan, India and Bangladesh and Others (Africa and eastern Europe)
Aldridge et al., 2016 [16]	16-44(94.4%)	Higher in Males (67.7%)	15 HTBCs (Bangladesh, Burkina Faso, Cambodia, Côte d’Ivoire, Eritrea, Ghana, Kenya, Laos, Niger, Pakistan, Somalia, Sudan, Tanzania, Thailand, and Togo)
Aldridge et al., 2016 [29]	≥ 65	Higher in females 116 (101–134) per 10^5^; Male: 79 (70–90) per 10^5^	15 HTBCs (Bangladesh, Burkina Faso, Cambodia, Côte d’Ivoire, Eritrea, Ghana, Kenya, Laos, Niger, Pakistan, Somalia, Sudan, Tanzania, Thailand, and Togo)
Dierberg et al., 2016 [24]	ND	Higher in males 77/92 (80.0%)	Tibet
Liu et al., 2016 [25]	ND	ND	Vietnam and Philippines
Ismail et al, 2018 [30]	ND	ND	Syria
Boogaard et al, 2020 [26]	≥18	Higher in males (58.7%)	Somalia and Eritrea

ND-Not Described.

## Discussion

The current systematic review summarized the magnitude of TB among refugees and migrants. The incidence of TB among refugees and migrants were ranged from 19 to 754 cases per 100,000 population among the seven included studies whereas the prevalence of TB among refugees and migrants were ranged from 18.7 to 535 cases per 100,000 population among the four included studies.

We found that more articles included in this review were from European countries in the year of publication 2016. This might be due to a large influx of refugees and migrants, mostly from high TB burden countries (HTBCs) of Africa and Asia, who moved mostly to Europe during this period [10, 31]. After the Syrian crisis in 2011, a remarkable increase in TB cases was reported in countries bordering Syria and also affected Europe [30].

The incidence and prevalence of TB among refugees and migrant populations varied among regions and across countries. The lowest incidence (19 cases per 100,000 population) was reported from the study done by Asadi et al., 2017 [22] in Alberta, Canada. This could be due to the study populations’ selection, as only 2%(5500) had previous history of TB and it is the fact that previous history of TB is the major determinant in the epidemiology of TB/MDR-TB [32–34]. However, the reported incidence of migrants was four times higher than the incidence (4.7/100,000 population) overall in the Alberta district in 2014 [22].

The highest incidence cases (754 cases per 100,000 population) were reported from the study done by Boogaard et al., 2020 from Eritrean and Somali refugees in Netherlands [26]. The incidence rate was much higher than the other included studies. A probable explanation for this finding is the additional risk for infection while traveling to Europe, where overcrowding and unsanitary conditions are common along travel routes. The other explanation could be case definition for incident TB case in which both pulmonary and extrapulmonary TB cases were included in the analysis that contribute to the high TB incidence rate in the asylum seekers.

The high incidence cases (431 cases per 100,000 population) were also reported from the study done by Dierberg et al., 2016 from Tibetan refugees in India [24]. This might be due to overcrowded living conditions of Tibetan refugees of whom more than half of TB cases in these refugees occurred in congregated settings. Moreover, use of the rapid diagnostic test and active case finding in the study might increase the detection of undiagnosed TB cases in congregate living settings. The reported incidence was more than two times higher compared to the overall incidence in India (181 cases per 100,000 population) in 2010 [24].

The lowest prevalence was reported from Syrian refugees (18.7 cases per 100,000 population) in Turkey [30]. This could be due to the lower TB prevalence reported by both Syria (23/100,000) and Turkey (22/100,000) before the onset of the Syrian crisis, which might result in low TB prevalence of the Syrian refugees in hosting country. After the beginning of the Syrian crisis in 2011, a remarkable increase in TB cases was reported in countries bordering Syria. Turkey is bordered by Syria and reported a noticeable increase in the proportion of imported TB cases from 1.3% in 2011 to 6.8% in 2015 [30].

The highest prevalence of 535 per 100 000 population was reported from asylum seekers in Bologna, northern Italy [27]. The prevalence found in migrants is 80 times greater compared to the estimated prevalence rate in Italy (6.7 per 100 000 population). Europe is the lowest TB burden region and the majority of reports indicated < 20/100,000 population [35]. The highest prevalence in this study compared to other findings in Europe may be explained by the different nature of migration flows and extended staying in detention and overcrowding for several months in Libya before reaching Italian coasts, which might increase the risk of TB transmission and the risk of progression from infection to disease. In contrast, the screening of migrants in a reception setting which underwent by adopting a chest radiograph with a specific TB questionnaire might overestimate the TB screening result, unlike the majority of European countries performing a chest radiograph based on active TB screening [27].

Article search was limited to only three electronic data bases: Google scholar, Medline/PubMed and Web of science. EMBASE and Scopus databases were not accessed due to free access restriction in the institutes where this review conducted.

## Conclusion

The current systematic review showed that the magnitude of TB among refugees and migrant populations was higher than in the host countries in the included studies. The findings revealed the need to implement and improve TB surveillance, promoting TB screening, TB prevention and control in refugees and migrant populations globally.

## Supporting information

S1 File PRISMA checklist. (PDF)

S2 File Study quality assessment. (PDF)

S3 File PROSPERO registration. (PDF)

## References

[1] Sakula A. Robert Koch: centenary of the discovery of the tubercle bacillus, 1882. Thorax. 1982;37(4):246–51. doi: 10.1136/thx.37.4.246 6180494 PMC459292

[2] Centers for Disease Control and Prevention. Tuberculosis: basic TB facts. Atlanta, GA: CDC. http://www.cdc…; 2012.

[3] World Health Organization. Global tuberculosis report 2020. WHO. Geneva, Switzerland.

[4] Connolly MiA, Gayer M, Ottmani S-E, World Health Organization. Tuberculosis care and control in refugee and displaced populations: an interagency field manual: Geneva: WHO; 2007.

[5] Jackson IC. The 1951 Convention Relating to the Status of Refugees: A universal basis for protection. Int’l J Refugee L. 1991;3:403.

[6] Urquia ML, Gagnon AJ. Glossary: migration and health. Journal of Epidemiology & Community Health. 2011;65(5):467–72. doi: 10.1136/jech.2010.109405 21282138

[7] Pavli A, Maltezou H. Health problems of newly arrived migrants and refugees in Europe. Journal of travel medicine. 2017;24(4). doi: 10.1093/jtm/tax016 28426115

[8] United Nation High Commisioner for Refugees. UNHCR’s annual Global Trends report. 2018.

[9] United Nation High Commisioner for Refugees. East and Horn of Africa 2018 end year report. 2018.

[10] Figueroa-Munoz JI, Ramon-Pardo P. Tuberculosis control in vulnerable groups. Bulletin of the World Health Organization. 2008;86:733–5. doi: 10.2471/blt.06.038737 18797650 PMC2649499

[11] Pareek M, Greenaway C, Noori T, Munoz J, Zenner D. The impact of migration on tuberculosis epidemiology and control in high-income countries: a review. BMC medicine. 2016;14(1):48. doi: 10.1186/s12916-016-0595-5 27004556 PMC4804514

[12] Rendon A, Centis R, Zellweger J-P, Solovic I, Torres-Duque C, Cordeiro CR, et al. Migration, TB control and elimination: whom to screen and treat. Pulmonology. 2018;24(2):99–105. doi: 10.1016/j.rppnen.2017.11.007 29292196

[13] Ejeta E, Beyene G, Balay G, Bonsa Z, Abebe G. Factors associated with unsuccessful treatment outcome in tuberculosis patients among refugees and their surrounding communities in Gambella Regional State, Ethiopia. PloS one. 2018;13(10):e0205468. doi: 10.1371/journal.pone.0205468 30335777 PMC6193657

[14] Legesse T, Admenur G, Gebregzabher S, Woldegebriel E, Fantahun B, Tsegay Y, et al. Tuberculosis (TB) in the refugee camps in Ethiopia: trends of case notification, profile, and treatment outcomes, 2014 to 2017. 2021;21(1):1–12. 10.1186/s12879-021-05828-y PMC7856765 33535974

[15] Lu CW, Lee YH, Pan YH, Chang HH, Wu YC, Sheng WH, et al. Tuberculosis among migrant workers in Taiwan. Globalization and Health. 2019;15(1):18. doi: 10.1186/s12992-019-0461-2 30819237 PMC6394038

[16] Aldridge RW, Zenner D, White PJ, Williamson EJ, Muzyamba MC, Dhavan P, et al. Tuberculosis in migrants moving from high-incidence to low-incidence countries: a population-based cohort study of 519 955 migrants screened before entry to England, Wales, and Northern Ireland. Lancet (London, England). 2016;388(10059):2510–8. doi: 10.1016/S0140-6736(16)31008-X 27742165 PMC5121129

[17] Sossou M-A. Refugees’ life transitions from displacement to durable resettlement. Handbook of stressful transitions across the lifespan: Springer; 2010. p. 465–84.

[18] World Health Organization. Consultation on Tuberculosis and Migration in the Western Pacific Region, Manila, Philippines, 26–27 March 2013: meeting report. Manila: WHO Regional Office for the Western Pacific; 2013.

[19] Liberati A, Altman DG, Tetzlaff J, Mulrow C, Gøtzsche PC, Ioannidis JP, et al. The PRISMA statement for reporting systematic reviews and meta-analyses of studies that evaluate health care interventions: explanation and elaboration. PLoS medicine. 2009;6(7):e1000100. doi: 10.1371/journal.pmed.1000100 19621070 PMC2707010

[20] Modesti PA, Reboldi G, Cappuccio FP, Agyemang C, Remuzzi G, Rapi S, et al. Panethnic differences in blood pressure in Europe: a systematic review and meta-analysis. PloS one. 2016;11(1):e0147601. doi: 10.1371/journal.pone.0147601 26808317 PMC4725677

[21] Collaboration C. Cochrane Consumers and Communication Review Group: data extraction template for Cochrane reviews. (Internet)(nd) Available from: cccrg cochrane org/author-resources. 2011.

[22] Asadi L, Heffernan C, Menzies D, Long R. Effectiveness of Canada’s tuberculosis surveillance strategy in identifying immigrants at risk of developing and transmitting tuberculosis: a population-based retrospective cohort study. The Lancet Public Health. 2017;2(10):e450–e7. doi: 10.1016/S2468-2667(17)30161-5 29253429

[23] Ospina JE, Orcau À, Millet J-P, Ros M, Gil S, Caylà JA, et al. Epidemiology of tuberculosis in immigrants in a large city with large-scale immigration (1991–2013). PloS one. 2016;11(10):e0164736. doi: 10.1371/journal.pone.0164736 27749904 PMC5066941

[24] Dierberg KL, Dorjee K, Salvo F, Cronin WA, Boddy JB, Cirillo D, et al. Improved detection of tuberculosis and multidrug-resistant tuberculosis among Tibetan refugees, India. Emerging infectious diseases. 2016;22(3):463. doi: 10.3201/eid2203.140732 26889728 PMC4766920

[25] Liu Y, Posey DL, Cetron MS, Painter JA. Effect of a culture-based screening algorithm on tuberculosis incidence in immigrants and refugees bound for the United States. Annals of internal medicine. 2015;162(6):420. doi: 10.7326/M14-2082 25775314 PMC4646057

[26] van den Boogaard J, Slump E, Schimmel HJ, van der Hoek W, van den Hof S, de Vries G. High Incidence of Active Tuberculosis in Asylum Seekers from Eritrea and Somalia in the First 5 Years after Arrival in the Netherlands. Emerging Infectious Diseases. 2020;26(4):675. doi: 10.3201/eid2604.190123 32186488 PMC7101101

[27] Vanino E, Tadolini M, Attard L, Po C, Francia F, Giannini A, et al. Systematic tuberculosis screening in asylum seekers in Italy. Clinical Infectious Diseases. 2017;65(8):1407–9. doi: 10.1093/cid/cix503 29017255

[28] Meier V, Artelt T, Cierpiol S, Gossner J, Scheithauer S. Tuberculosis in newly arrived asylum seekers: A prospective 12 month surveillance study at Friedland, Germany. International journal of hygiene and environmental health. 2016;219(8):811–5. doi: 10.1016/j.ijheh.2016.07.018 27498629

[29] Aldridge RW, Zenner D, White PJ, Muzyamba MC, Loutet M, Dhavan P, et al. Prevalence of and risk factors for active tuberculosis in migrants screened before entry to the UK: a population-based cross-sectional study. The Lancet Infectious Diseases. 2016;16(8):962–70. doi: 10.1016/S1473-3099(16)00072-4 27013215

[30] Ismail MB, Rafei R, Dabboussi F, Hamze M. Tuberculosis, war, and refugees: spotlight on the Syrian humanitarian crisis. PLoS pathogens. 2018;14(6):e1007014. doi: 10.1371/journal.ppat.1007014 29879218 PMC5991642

[31] Trends G. Forced Displacement in 2015. UNHCR, The UN Refugee Agency, Geneva, Switzerland. 2016.

[32] Sambas MFMK, Rabbani U, Al-Gethamy MMM, Surbaya SH, Alharbi FFI, Ahmad RGA, et al. Prevalence and determinants of multidrug-resistant tuberculosis in Makkah, Saudi Arabia. 2020;13:4031. doi: 10.2147/IDR.S277477 33204120 PMC7667158

[33] Girum T, Muktar E, Lentiro K, Wondiye H, Shewangizaw MJTd, travel medicine, vaccines. Epidemiology of multidrug-resistant tuberculosis (MDR-TB) in Ethiopia: a systematic review and meta-analysis of the prevalence, determinants and treatment outcome. 2018;4(1):1–12. 10.1186/s40794-018-0065-5 PMC6000958 29942536

[34] Lomtadze N, Aspindzelashvili R, Janjgava M, Mirtskhulava V, Wright A, Blumberg H, et al. Prevalence and risk factors for multidrug-resistant tuberculosis in the Republic of Georgia: a population-based study. 2009;13(1):68–73. PMC2645031 19105881

[35] World Health Organization. European Centre for Disease Prevention and Control. Tuberculosis Surveillance and monitoring in Europe. 2014.

