# Supplementary material for: Tuberculosis among refugees and migrant populations: Systematic review
Source: PLoS One. 2022 Jun 9;17(6):e0268696. doi: 10.1371/journal.pone.0268696 (PMC9182295; doi:10.1371/journal.pone.0268696)
Supplement: S2 File — (PDF) [file pone.0268696.s002.pdf]

Supplementary Information. Study quality assessment

Nr.	Author, year	Selection score	Comparability score	Outcome score	Final Quality score	Authors for decision
1.	Lu et al., 2019	4 star	1 star	3 star	Good	AM, HH
2.	Asadi et al., 2017	4 star	1 star	3 star	Good	AM, HH
3.	Vanino et al., 2017	1 star	1 star	2 star	Poor	AM, HH
4.	Meir et al., 2016	2 star	1 star	3 star	Fair	KE
5.	Ospinia et al., 2016	5 star	1 star	3 star	Good	AM, HH
6.	Aldridge et al., 2016	5 star	2 star	3 star	Good	AM, HH
7.	Aldridge et al., 2016	5 star	1 star	3 star	Good	AM, HH
8.	Dierberg et al., 2016	2 star	3 star	2 star	Fair	AM, HH
9.	Liu et al., 2016	2 star	1 star	3 star	Fair	AM, HH
10.	Ismail et al., 2018	0 star	0 star	1 star	Poor	AM, HH
11.	Boogaard et al, 2020	4 star	1 star	2 star	Good	KE

Good quality study was scored 3 or 4 stars in participant selection, 1 or 2 stars in comparability of groups, and 2 or 3 stars in outcome(s) assessment.

Fair quality study was scored 2 stars in participant selection, 1 or 2 stars in comparability of the groups, and 2 or 3 stars in outcome(s) assessment.

Poor quality study was scored 0 or 1 star in participant selection, 0 or 1 star in comparability of the groups and 0 or 1 star in outcome(s) assessment.

In case of disparity between the two authors (AM and HH) during study selection process, the disparities were resolved by the decision of the second author (GD).
