# Supplementary material for: Tuberculosis among refugees and migrant populations: Systematic review
Source: PLoS One. 2022 Jun 9;17(6):e0268696. doi: 10.1371/journal.pone.0268696 (PMC9182295; doi:10.1371/journal.pone.0268696)
Supplement: S3 File — (PDF) [file pone.0268696.s003.pdf]

PROSPERO

International prospective register of systematic reviews

Burden of Tuberculosis among refugees and Migrant populations: a systematic review.

Abyot Meaza Dasho, Habteyes Hailu Tolla

To enable PROSPERO to focus on COVID-19 registrations during the 2020 pandemic, this registration record was automatically published exactly as submitted. The PROSPERO team has not checked eligibility.

Citation

Abyot Meaza Dasho, Habteyes Hailu Tolla. Burden of Tuberculosis among refugees and Migrant populations: a systematic review. PROSPERO 2020 CRD42020157619 Available from: https://www.crd.york.ac.uk/prospero/display_record.php?ID=CRD42020157619

Review question

What are the populations mostly affected by Tuberculosis among refugees

What is the epidemiology of Tuberculosis among Refugees and migrant populations

What is the Prevalence/Incidence of Tuberculosis among Refugees and migrant populations

Searches

We will search the electronic databases Web of Sciences, PubMed/MEDLINE, Scopus, Embase,

Complementary

Index and Academic Search Complete for English language articles published between 2014 and 2019.

November 21, 2019

Types of study to be included

Systematic review. Epidemiological studies and surveillance studies included in the review

Condition or domain being studied

Tuberculosis (TB) is an increasingly important cause of morbidity and mortality among refugee and migrant populations. Refugees and Migrants are among the most vulnerable populations at increased risk of developing TB and of poor access to TB care and control services. Factors including malnutrition, overcrowding, poor living condition, their mobile nature and poor access to health care system increase the vulnerability of these populations. Conflict, poverty, limited resource, political instability and unemployment are the most common cause of large population displacement.

Participants/population

Refugees, persons who are outside their country and cannot return owing to a well-founded fear of persecution because of their race, religion, nationality, political opinion or membership in a particular social group

Migrant populations

Intervention(s), exposure(s)

Tuberculosis

Comparator(s)/control

N/A

Main outcome(s)

The burden of Tuberculosis in the refugee and migrant populations will be reviewed. The main outcomes will be Incidence, prevalence and mortality of Tuberculosis in the five years publication period will be reviewed. We included Tuberculosis Epidemiological studies(Incidence, Prevalence, Survey, notifications) in refugee and migrant populations since 2005 and reported in all age groups.

* Measures of effect

Not applicable

Additional outcome(s)

Tuberculosis Notifications rates and among refugees and migrant populations will be reviewed. The impact of Refugees and migration also reviewed. Factors including malnutrition, overcrowding, poor living condition, their mobile nature and poor access to health care system increase the vulnerability of these populations. Conflict, poverty, limited resource, political instability and unemployment are the most common cause of large population displacement.

Data extraction (selection and coding)

We included Tuberculosis Epidemiological studies (Incidence, Prevalence, Survey, notifications) in refugee and migrant populations since 2005 and reported in all age groups. We excluded before 2014.

Risk of bias (quality) assessment

The quality of the studies selected was assessed independently by two authors (AMD and HHT) using Crombie's tool, which contains seven items for quality assessment. These items were assessed for appropriateness of design, adequacy of data description, representativeness of the total sample, clarity, reliability and validity of the measurements, statistical significance, appropriateness and adequacy of the analyses. The quality of each article was then scored accordingly.

Strategy for data synthesis

Data entry will be done using SPSS version 23 software packages and epidata. Double data entry will be also done using online OpenClinica for data entry and cleaning.

Analysis of subgroups or subsets

Data analysis will be done using STATA ver 14 and results will be interpreted based on 95% confidence

interval, statistically significant if p-value <0.05.

Contact details for further information

Abyot Meaza Dasho

abimeaza@gmail.com

Organizational affiliation of the review

Ethiopian Public Health Institute

WWW.ephi.gov.et

Review team members and their organizational affiliations

Mr Abyot Meaza Dasho. Ethiopian Public Health Institute

Mr Habteyes Hailu Tolla. Ethiopian Public Health Institute

Type and method of review

Systematic review

Anticipated or actual start date

18 November 2019

Anticipated completion date

31 December 2019

Funding sources/sponsors

Addis Ababa University and Ethiopian Public Health Institute

Conflicts of interest

Language

English

Country

Ethiopia

Stage of review

Review Ongoing

Subject index terms status

Subject indexing assigned by CRD

Subject index terms

MeSH headings have not been applied to this record

Date of registration in PROSPERO

10 July 2020

Date of first submission

07 November 2019

Stage of review at time of this submission

Stage Started Completed

Stage	Started	Completed
Preliminary searches	Yes	No
Piloting of the study selection process	No	No
Formal screening of search results against eligibility criteria	Yes	No
Data extraction	No	No
Risk of bias (quality) assessment	No	No
Data analysis	No	No

The record owner confirms that the information they have supplied for this submission is accurate and complete and they understand that deliberate provision of inaccurate information or omission of data may be construed as scientific misconduct. The record owner confirms that they will update the status of the review when it is completed and will add publication details in due course.

Versions

10 July 2020

PROSPERO

This information has been provided by the named contact for this review. CRD has accepted this information in good faith and registered the review in PROSPERO. The registrant confirms that the information supplied for this submission is accurate and complete. CRD bears no responsibility or liability for the content of this registration record, any associated files or external websites. Powered by TCPDF (www.tcpdf.org)
