# Supplementary material for: Tuberculosis among refugees and migrant populations: Systematic review
Source: PLoS One. 2022 Jun 9;17(6):e0268696. doi: 10.1371/journal.pone.0268696 (PMC9182295; doi:10.1371/journal.pone.0268696)
Supplement: Attachment — Submitted filename: PONE-D-21-33712 revised.pdf [file pone.0268696.s004.pdf]

PLOS ONE

Tuberculosis among refugees and migrant populations: systematic review.

--Manuscript Draft--

**Manuscript Number:**	PONE-D-21-33712
**Article Type:**	Research Article
**Full Title:**	Tuberculosis among refugees and migrant populations: systematic review.
**Short Title:**	TB among refugees and migrants
**Corresponding Author:**	Abyot Meaza<br>Ethiopian Public Health Institute<br>Addis Ababa, ETHIOPIA
**Keywords:**	Tuberculosis, refugees and migrants
**Abstract:**	Tuberculosis (TB) is an important cause of morbidity and mortality among refugees and migrant populations. These groups are among the most vulnerable populations at increased risk of developing TB. However, there is a lack of systematic review that attempts to summarize TB among refugees and migrant populations. The present systematic review aimed to summarize evidence on the magnitude of TB among refugees and migrant populations. A systematic search was done to retrieve the articles published from 2014 to 2020 in English language from electronic databases. Key searching terms were used in both free text and Medical Subject Heading (MeSH). Articles which had reported the magnitude of TB among refugees and migrant populations were included in the review. We assessed the risk of bias, and quality of the included studies with a modified version of the Newcastle–Ottawa Scale (NOS). Included studies which had reported incidence or prevalence data were eligible for data synthesis. The results were shown as summary tables.<br>The incidence and prevalence of TB ranged from 19 to 754 cases per 100,000 population and 18.7 to 535 cases per 100,000 population respectively among the included studies. Article search was limited to only three electronic data bases due to free access restriction in the institutes where this review conducted. The current finding shows that the incidence and prevalence of TB among refugees and migrant populations is higher than in the host countries. This implies the need to implement and improve TB prevention and control in refugees and migrant populations globally.<br>No funding source was available in the present review and the protocol of this review was registered on PROSPERO (International prospective register of systematic reviews) with ID number, CRD42020157619.
**Order of Authors:**	Abyot Meaza
	Habteyes Hailu Tola
	Kirubel Eshetu
	Girmay Medhin
	Balako Gumi
**Additional Information:**	
**Question**	**Response**
**Financial Disclosure**<br><br>Enter a financial disclosure statement that describes the sources of funding for the work included in this submission. Review the submission guidelines for detailed requirements. View published research articles from *PLOS ONE* for specific examples.	The author(s) received no specific funding for this work

Powered by Editorial Manager® and ProduXion Manager® from Aries Systems Corporation

This statement is required for submission and **will appear in the published article** if the submission is accepted. Please make sure it is accurate.<br><br>**Unfunded studies**<br>Enter: *The author(s) received no specific funding for this work.*<br><br>**Funded studies**<br>Enter a statement with the following details:<br>• Initials of the authors who received each award<br>• Grant numbers awarded to each author<br>• The full name of each funder<br>• URL of each funder website<br>• Did the sponsors or funders play any role in the study design, data collection and analysis, decision to publish, or preparation of the manuscript?<br>• **NO** - Include this sentence at the end of your statement: *The funders had no role in study design, data collection and analysis, decision to publish, or preparation of the manuscript.*<br>• **YES** - Specify the role(s) played.<br><br>* typeset	
**Competing Interests**<br><br>Use the instructions below to enter a competing interest statement for this submission. On behalf of all authors, disclose any competing interests that could be perceived to bias this work—acknowledging all financial support and any other relevant financial or non-financial competing interests.<br><br>This statement is required for submission and **will appear in the published article** if the submission is accepted. Please make sure it is accurate and that any funding sources listed in your Funding Information later in the submission form are also declared in your Financial Disclosure statement.<br><br>View published research articles from *PLOS ONE* for specific examples.	The author(s) declare that there is no any conflict of interest that might influence this work.

**NO authors have competing interests**<br><br>Enter: *The authors have declared that no competing interests exist.*<br><br>**Authors with competing interests**<br><br>Enter competing interest details beginning with this statement:<br><br>*I have read the journal's policy and the authors of this manuscript have the following competing interests: [insert competing interests here]*<br><br>* typeset	
**Ethics Statement**<br><br>Enter an ethics statement for this submission. This statement is required if the study involved:<br><br>• Human participants<br>• Human specimens or tissue<br>• Vertebrate animals or cephalopods<br>• Vertebrate embryos or tissues<br>• Field research<br><br>Write "N/A" if the submission does not require an ethics statement.<br><br>General guidance is provided below. Consult the submission guidelines for detailed instructions. **Make sure that all information entered here is included in the Methods section of the manuscript.**	N/A, as this review was based on previously published articles.

**Format for specific study types**<br><br>**Human Subject Research (involving human participants and/or tissue)**<br>• Give the name of the institutional review board or ethics committee that approved the study<br>• Include the approval number and/or a statement indicating approval of this research<br>• Indicate the form of consent obtained (written/oral) or the reason that consent was not obtained (e.g. the data were analyzed anonymously)<br><br>**Animal Research (involving vertebrate animals, embryos or tissues)**<br>• Provide the name of the Institutional Animal Care and Use Committee (IACUC) or other relevant ethics board that reviewed the study protocol, and indicate whether they approved this research or granted a formal waiver of ethical approval<br>• Include an approval number if one was obtained<br>• If the study involved *non-human primates*, add *additional details* about animal welfare and steps taken to ameliorate suffering<br>• If anesthesia, euthanasia, or any kind of animal sacrifice is part of the study, include briefly which substances and/or methods were applied<br><br>**Field Research**<br><br>Include the following details if this study involves the collection of plant, animal, or other materials from a natural setting:<br>• Field permit number<br>• Name of the institution or relevant body that granted permission	
**Data Availability**<br><br>Authors are required to make all data underlying the findings described fully available, without restriction, and from the time of publication. PLOS allows rare exceptions to address legal and ethical concerns. See the PLOS Data Policy and FAQ for detailed information.	Yes - all data are fully available without restriction

A Data Availability Statement describing where the data can be found is required at submission. Your answers to this question constitute the Data Availability Statement and **will be published in the article**, if accepted.<br><br>**Important:** Stating 'data available on request from the author' is not sufficient. If your data are only available upon request, select 'No' for the first question and explain your exceptional situation in the text box.<br><br>Do the authors confirm that all data underlying the findings described in their manuscript are fully available without restriction?	
**Describe where the data may be found in full sentences. If you are copying our sample text, replace any instances of XXX with the appropriate details.**<br><br>• If the data are **held or will be held in a public repository**, include URLs, accession numbers or DOIs. If this information will only be available after acceptance, indicate this by ticking the box below. For example: *All XXX files are available from the XXX database (accession number(s) XXX, XXX.).*<br>• If the data are all contained **within the manuscript and/or Supporting Information files**, enter the following: *All relevant data are within the manuscript and its Supporting Information files.*<br>• If neither of these applies but you are able to provide **details of access elsewhere**, with or without limitations, please do so. For example:<br><br>*Data cannot be shared publicly because of [XXX]. Data are available from the XXX Institutional Data Access / Ethics Committee (contact via XXX) for researchers who meet the criteria for access to confidential data.*<br><br>*The data underlying the results presented in the study are available from (include the name of the third party*	All data and materials that support the final result are available.

and contact information or URL).<br>• This text is appropriate if the data are owned by a third party and authors do not have permission to share the data.<br><br>* typeset	
Additional data availability information:	Tick here if the URLs/accession numbers/DOIs will be available only after acceptance of the manuscript for publication so that we can ensure their inclusion before publication.

Powered by Editorial Manager® and ProduXion Manager® from Aries Systems Corporation

Manuscript Click here to access/download;Manuscript;Manuscript.docx

Tuberculosis among refugees and migrant populations: systematic review.

Abyot Meaza[1, 2], Habteyes Hailu Tola[1], Kirubel Eshetu[1], Girmay Medhin[2], Balako Gumi[2]

Affiliations: [1] Ethiopian Public Health Institute (EPHI)

[2] Addis Ababa University, Aklilu Lemma Institute of Pathobiology.

[1]Abyot Meaza, Ethiopian Public Health Institute, email: abimeaza@gmail.com, Addis Ababa, Ethiopia, Corresponding Author

[1]Habteyes Hailu Tola, Ethiopian Public Health Institute, email: habtetola@gmail.com, Addis Ababa, Ethiopia

[1]Kirubel Eshetu, Ethiopian Public Health Institute, email: kirub1625@gmail.com, Addis Ababa, Ethiopia

[2]Girmay Medhin, Addis Ababa University, Aklilu Lemma Institute of Pathobiology, email: girmay.medhin@aau.edu.et , Addis Ababa, Ethiopia

[2]Balako Gumi, Addis Ababa University, Aklilu Lemma Institute of Pathobiology, email: balako.gumi@aau.edu.et , Addis Ababa, Ethiopia

[*]Corresponding Author: Abyot Meaza, National TB Reference Laboratory (NTRL), EPHI, Swaziland Street, PO Box 1242, Addis Ababa, Ethiopia. Email: abimeaza@gmail.com

Abstract

Tuberculosis (TB) is an important cause of morbidity and mortality among refugees and migrant populations. These groups are among the most vulnerable populations at increased risk of developing TB. However, there is a lack of systematic review that attempts to summarize TB among refugees and migrant populations. The present systematic review aimed to summarize evidence on the magnitude of TB among refugees and migrant populations. A systematic search was done to retrieve the articles published from 2014 to 2021 in English language from electronic databases. Key searching terms were used in both free text and Medical Subject Heading (MeSH). Articles which had reported the magnitude of TB among refugees and migrant populations were included in the review. We assessed the risk of bias, and quality of the included studies with a modified version of the Newcastle–Ottawa Scale (NOS). Included studies which had reported incidence or prevalence data were eligible for data synthesis. The results were shown as summary tables.

The incidence and prevalence of TB ranged from 19 to 754 cases per 100,000 population and 18.7 to 535 cases per 100,000 population respectively among the included studies. Article search was limited to only three electronic data bases due to free access restriction in the institutes where this review conducted. The current finding shows that the incidence and prevalence of TB among refugees and migrant populations is higher than in the host countries. This implies the need to implement and improve TB prevention and control in refugees and migrant populations globally.

No funding source was available in the present review and the protocol of this review was registered on PROSPERO (International prospective register of systematic reviews) with ID number, CRD42020157619.

Key words: Tuberculosis, refugees and migrants

Introduction

Tuberculosis (TB) is an infectious disease caused by the bacillus *Mycobacterium tuberculosis* (1). It typically affects the lungs (pulmonary TB) but can also affect other sites (extra pulmonary TB) (2). TB is causing morbidity and mortality each year among millions across the world. It is the tenth leading cause of death worldwide and the leading cause of a single infectious agent (3). The WHO recent report indicated 10 million new cases and 1.4 million deaths occurred globally (3).

Tuberculosis is an important cause of morbidity and mortality among refugees and migrant populations (4). Refugees are defined as people who are outside their country and cannot return due to a well-founded fear of persecution because of their race, religion, nationality, political opinion or membership in a particular social group (5). Whereas migrants are defined as people and/or family members, moving to another country or region to better their material or social conditions and improve the prospects for themselves or their family (6).

The number of international migrants reached 244 million in 2015, with almost two-thirds travelling to industrialized countries(7). The number of refugees also exceeded 70 million in 2018, which was the highest level that the United Nation High Commission for Refugees (UNHCR) has seen in its almost 70 years (8). Sub-Saharan Africa hosted more than 26% of the world's refugee population, and the number of refugees in the Horn of Africa exceeded 3 million, with Uganda (1.1 million) and Ethiopia (905,000) hosting the largest numbers (9).

Refugees and migrants are the most affected populations at risk of developing TB and other infectious diseases due to their living conditions (7). Poor living conditions and overcrowding in refugee settlements potentially increase the risk of TB infection (10). The impact of migration on TB epidemiology is high (11). The arrival of large groups of refugees can affect TB control efforts in receiving countries by significantly increasing the disease burden and cost of health services (10). Since the majority of the migrants originate from low-income countries with a high TB burden, it imposes new challenges for global TB control and eradication towards achieving the stop TB strategy (12).

Although there is published evidence in different geographical area[illegible]here is a lack of systematic review that attempts to summarize the magnitude of TB among refugees and migrant populations. The present systematic review aimed to summarize existing published literature on the magnitude (incidence and prevalence) of TB among refugees and migrant populations. The objective of this systematic review is to review the magnitude of TB among refugees and migrant populations living in refugee camps and in migrant-hosting countries.

Methods

Search strategy

We conducted a systematic review of published articles and summarized findings about the incidence and prevalence of TB among refugees and migrant populations living in refugee camps and in migrant-hosting countries following PRISMA (Preferred Reporting Items for Systematic Reviews and Meta-Analyses) standards (13).

We systematically searched electronic databases: Medline/PubMed, Web of Science and Google scholar for English language articles. We used sensitive searching method to include all potential articles in the data bases. The search was restricted to papers published in the last 7 years (between 2014 and 2021). The electronic database search was conducted from 01 August 2021 to 4 November 2021. We used a search strategy by combining key-terms: "Tuberculosis", "Multidrug-Resistant", "Pulmonary Tuberculosis", "Pleural Tuberculosis", "Miliary Tuberculosis", "Meningeal Tuberculosis", "Lymph node Tuberculosis", "Laryngeal Tuberculosis", "Mycobacterium tuberculosis", "Prevalence", "Epidemiology", "Cross-Sectional Studies", "Incidence", "Cohort Studies", "mortality", "Refugees", "Refugee Camps", "Migrant" and "Transients and Migrants" both in MeSH and free text terms. Two authors (AM and HH) independently reviewed the titles, abstracts and full articles of retrieved studies.

Inclusion and Exclusion criteria

We included cross-sectional studies, retrospective cohort studies and cohort studies which had reported incidence or prevalence as an outcome variable. Eligible studies which were conducted in different parts of the world and published from 2014 to 2021 were included in the review. We excluded studies with different outcome variables, and studies published in non-English languages. The included studies were grouped as incidence and prevalence for the syntheses.

Study selection process

A total of 1241 records were identified from data base searches and 1188 articles were excluded after title screening due to outcome variable difference with the present review objective. Upon title and abstract screening of 53 articles, 31 articles were excluded based on the exclusion criteria. Then the remaining 22 articles in full text were reviewed for eligibility and 12 articles were excluded. In the final grade, 10 articles remained to be included in the review and one

article was also selected from the bibliography of the included study. Thus, a total of 11 studies were included in this systematic review (Figure 1). Eligibility assessment was performed independently in an unblinded standardized manner by two reviewers (AM & HH) and disagreements between reviewers were resolved by discussion.

Study quality assessment

We assessed the risk of bias, certainty and quality of the included studies with a modified version of the Newcastle–Ottawa Scale(14). The scale assesses three key points of a given study: participant selection; comparability of the groups; and outcome (s) assessment. We assigned stars for each point of the scale to categorize the studies into good, fair and poor quality based on the NOS (14). A good quality study was scored 3 or 4 stars in participant selection, 1 or 2 stars in comparability of groups, and 2 or 3 stars in outcome (s) assessment. A fair quality study was scored 2 stars in participant selection, 1 or 2 stars in comparability of the groups, and 2 or 3 stars in outcome (s) assessment. A poor-quality study was scored 0 or 1 star in participant selection, 0 stars in comparability of the groups and 0 or 1 star in outcome (s) assessment. In the case of disparity between the two authors (AM and HH) during the study selection process, the disparities were resolved by the decision of the second author (KE).

Data extraction

We developed a data extraction format based on the Cochrane Consumers and Communication Review Group's data extraction template (15). The template was pilot-tested on 3 randomly-selected included studies, and refined accordingly. One review author [AM] extracted the data from included studies and the second author [HH] checked the extracted data. Disagreements were resolved by discussion between the two authors and a third author [KE]. We extracted

information on characteristics of participants such as age range, mean of age, sex ratio and county of origin of refugees and migrant populations. Study characteristics such as first author and year of publication; study location; study population; design; sample size; year (s) data were collected; TB diagnosis method; reported burden of TB (incidence, and prevalence per 100 000 population).

Ethics consideration

Ethical approval was not required, as this review was based on previously published articles. However, the protocol of this review was registered on PROSPERO, University of York, Centre for Reviews and Dissemination and can be accessed with ID number CRD42020157619.

Statistical analysis

Descriptive statistics were used to analyze the extracted data from included studies. Age range, mean age, sex ratio and the most reported countries of origin in TB cases among refugees and migrants were summarized to describe study characteristics. The reported burden of TB (incidence and prevalence) was summarized in a table. TB incidence and/or prevalence were calculated if the number of TB cases and population estimates were provided. Included studies which had reported the above data were eligible for data synthesis. Sensitivity analysis was conducted and shown as summary tables. Risk of bias due to missing results were minimized by inclusion of results from sources.

Results

[illegible]ven articles published from studies conducted in 10 countries and addressed TB in migrants, refugees, and asylum seekers. Nine articles were published on refugees and migrants in Europe, three studies in Asia, one study in the United States of America and one in Canada. Half of the included articles (6/11) were published in 2016. The country of origin of refugees and migrants screened for TB varied widely across regions and countries. The design and scope of the studies varied across the included studies. Six cross sectional studies, three retrospective cohort studies, one cohort study and one study with a scientific report were included in the present review(Tab[illegible] 1 and 2). We observed heterogeneity among results of the included studies by grouping the study results in to incidence and prevalence.

Table 1. Study characteristics and TB Incidence results among the included studies.

First author, year of publication	Study location	Design	Study population	Sample size	Year(s) data collected	Type of TB diagnosis	Incidence
**Lu et al., 2019(16)**	Taiwan	Retrospective Cohort	Migrants	379422	2004 to 2013	CXR	58.4 per 10^5<br>*Taiwan: 45.5–76.8 per 10^5*
**Asadi et al., 2017(17)**	Alberta, Canada	Retrospective cohort	Migrants and refugees	223225	2002 to 2013	Culture	$19/10^5$<br>Alberta: $4.7/10^5$
**Ospinia et al., 2016(18)**	Spain	Cross sectional	Immigrants	3284	1991 to 2013	CXR and Bacteriologic	$105.9/10^5$<br>Highest Pakistan, India, Bangladesh ($675/10^5$), followed by Africa ($329/10^5$)
**Aldridge et al., 2016(19)**	England, Wales & N. Ireland	Cohort	Migrants	519955	2006 to 2012	CXR and Bacteriologic	$147/10^5$

**Dierberg et al., 2016(20)**	India	Cross sectional	Refugees	27714	September 2011 to March 2013	CXR, Bacteriologic and Xpert MTB/RIF	431 cases/10^5 *India: 181 cases/10^5*
**Liu et al., 2016(21)**	USA	Cross sectional	Immigrants and refugees	1561460	2007 to 2012	Culture based examination	258 cases per 10^5
**Boogaard et al, 2020(22)**	Netherlands	retrospective cohort	Asylum seekers	26,057	January, 2013, to December 2017	Clinical, CXR, Bacteriologic	754 cases per 10^5 *Netherlands: 4.6/10^5*

Table 2. Study characteristics and TB prevalence results among the included studies.

First author, year of publication	Study location	Design	Study population	Sample size	Year(s) data collected	Type of TB diagnosis	Prevalence
**Vanino et al., 2017(23)**	Bologna, northern Italy	Cross sectional	Migrants	3366	2014 to 2015	CXR	535/10^5 *Italy: 6.7/10^5*
**Meir et al., 2016(24)**	Friedland, Germany	Cross sectional	Asylum seekers	11773	b/n 2014 and2015	Clinical, CXR and Bacteriologic	93/10^5 *Germany: 5.3/10^5*
**Aldridge et al., 2016(25)**	UK	Cross sectional	Migrants	476455	between 2005 and 2013	Bacteriologic confirmation	92 per 10^5
**Ismail et al, 2018(26)**	Turkey	Scientific report	Refugees	10689	ND	ND	18.7/10^5

ND-Not Described

More than 3 million refugees and migrants were screened for TB using history of TB, chest X-Ray (CXR), immunological and bacteriological examination mainly in hospital and reception settings. Ten studies collected data between 2002 and 2017, while one study collected data between 1991 and 2013 (Table 1 and 2).

Eleven studies have reported the incidence or prevalence of TB in refugees and migrant populations. The incidence of TB ranged from 19 to 754 cases per 100,000 population among the seven included studies (Table 1) and the prevalence of TB ranged from 18.7 to 535 cases per 100,000 population among the four included studies (Table 2).

More than half of the studies had reported TB cases whose age ranges were between 13 to 54 years and males were higher in reported TB cases as compared to females. The most reported countries of origin in TB cases among refugees and migrants were from Asia and Africa. India, Philippines, Pakistan, Syria, Thailand and Vietnam were the most reported countries of origin in TB cases from Asia, whereas Somalia and Eretria were the most reported countries in TB cases from Africa (Table3).

Table 3. Age range, gender and country of origin of notified TB cases across the included studies

First author, year of publication	Age range of TB cases	Male: Female ratio of TB cases	Country of origin
Lu et al., 2019(16)	45–54	Higher in males (3:1)	Indonesia, Philippines, Thailand, and Vietnam
Asadi et al., 2017(17)	13-37	Higher in males	Philippines, India, or China
Vanino et al., 2017(23)	18-41	All are males (18/18 males)	West Africa and India
Meir et al., 2016(24)	Mean age 31.2Yrs	ND	Eritrea, Russia, Pakistan and Syria

**Ospinia et al., 2016(18)**	25–44 years	Highest in Males (66.7%)	Latin America, Pakistan, India and Bangladesh and Others (Africa and eastern Europe)
**Aldridge et al., 2016(19)**	16-44(94.4%)	Higher in Males (67.7%)	15 HTBCs (Bangladesh, Burkina Faso, Cambodia, Côte d'Ivoire, Eritrea, Ghana, Kenya, Laos, Niger, Pakistan, Somalia, Sudan, Tanzania, Thailand, and Togo)
**Aldridge et al., 2016(25)**	≥ 65	Higher in females116 (101–134) per 10^5; Male: 79 (70–90) per 10^5	15 HTBCs (Bangladesh, Burkina Faso, Cambodia, Côte d'Ivoire, Eritrea, Ghana, Kenya, Laos, Niger, Pakistan, Somalia, Sudan, Tanzania, Thailand, and Togo)
**Dierberg et al., 2016(20)**	ND	Higher in males77/92 (80.0%)	Tibet
**Liu et al., 2016(21)**	ND	ND	Vietnam and Philippines
**Ismail et al, 2018(26)**	ND	ND	Syria
**Boogaard et al, 2020(22)**	≥ 18	Higher in males (58.7%)	Somalia and Eritrea

ND-Not Described

Discussion

The current systematic review summarized the magnitude of TB among refugees and migrants. The incidence of TB among refugees and migrants were ranged from 19 to 754 cases per 100,000 population among the seven included studies whereas the prevalence of TB among refugees and migrants were ranged from 18.7 to 535 cases per 100,000 population among the four included studies.

We found the most recent articles included in this review were from European countries in the year of publication 2016. This might be due to a large influx of refugees and migrants, mostly from high TB burden countries (HTBCs) of Africa and Asia, who moved mostly to Europe

during this period (10, 27). After the Syrian crisis in 2011, a remarkable increase in TB cases was reported in countries bordering Syria and also affected Europe (26).

The incidence and prevalence of TB among refugees and migrant populations varied among regions and across countries. The lowest incidence (19 cases per 100,000 population) was reported from the study done by Asadi et al., 2017 (28) in Alberta, Canada. This could be due to the study populations' selection, as only 5500 (2%) of those referred for TB surveillance previously had a history of TB. However, the reported incidence of migrants was four times higher than the incidence (4.7/100,000 population) overall in the Alberta district in 2014 (28).

The highest incidence cases (754 cases per 100,000 population) were reported from the study done by Boogaard et al., 2020 from Eritrean and Somali refugees in Netherlands (22). The incidence rate was much higher than the other included studies. A probable explanation for this finding is the additional risk for infection while traveling to Europe, where overcrowding and unsanitary conditions are common along travel routes. The other explanation could be case definition for incident TB case in which both pulmonary and extrapulmonary TB cases were included in the analysis that contribute to the high TB incidence rate in the asylum seekers.

The high incidence cases (431 cases per 100,000 population) were also reported from the study done by Dierberg et al., 2016 from Tibetan refugees in India(20). This might be due to overcrowded living conditions of Tibetan refugees of whom more than half of TB cases in these refugees occurred in congregated settings. Moreover, use of the rapid diagnostic test and active case finding in the study might increase the detection of undiagnosed TB cases in congregate living settings. The reported incidence was more than two times higher compared to the overall incidence in India (181 cases per 100,000 population) in 2010 (20).

The lowest prevalence was reported from Syrian refugees (18.7 cases per 100,000 population) in Turkey (26). This could be due to the lower TB prevalence reported by both Syria (23/100,000) and Turkey (22/100,000) before the onset of the Syrian crisis, which might result in low TB prevalence of the Syrian refugees in hosting country. After the beginning of the Syrian crisis in 2011, a remarkable increase in TB cases was reported in countries bordering Syria. Turkey is bordered by Syria and reported a noticeable increase in the proportion of imported TB cases from 1.3% in 2011 to 6.8% in 2015 (26).

The highest prevalence of 535 per 100 000 population was reported from asylum seekers in Bologna, northern Italy (23). The prevalence found in migrants is 80 times greater compared to the estimated prevalence rate in Italy (6.7 per 100 000 population). Europe is the lowest TB burden region and the majority of reports indicated < 20/100,000 population(29). The highest prevalence in this study compared to other findings in Europe may be explained by the different nature of migration flows and extended staying in detention and overcrowding for several months in Libya before reaching Italian coasts, which might increase the risk of TB transmission and the risk of progression from infection to disease. In contrast, the screening of migrants in a reception setting which underwent by adopting a chest radiograph with a specific TB questionnaire might overestimate the TB screening result, unlike the majority of European countries performing a chest radiograph based on active TB screening (23).

Article search was limited to only three electronic data bases: Google scholar, Medline/PubMed and Web of science. EMBASE and Scopus databases were not accessed due to free access restriction in the institutes where this review conducted.

The current systematic review showed that the magnitude of TB among refugees and migrant populations was higher than in the host countries in the included studies. The findings revealed the need to implement and improve TB surveillance, promoting TB screening, TB prevention and control in refugees and migrant populations globally.

Acknowledgments

We thank the Ethiopian Public Health Institute and Addis Ababa University.

Contributors

AM designed and carried out the systematic review and wrote the manuscript. HH participated in the data extraction and study quality appraisal. KE is involved in study quality appraisal. GM and BG advised and reviewed the systematic review.

Availability of data and materials

All data and materials that support the final result are available.

Competing interest

We declare that there is no any conflict of interest.

References

1. Sakula A. Robert Koch: centenary of the discovery of the tubercle bacillus, 1882. Thorax. 1982;37(4):246-51.

2. Centers for Disease Control and Prevention. Tuberculosis: basic TB facts. Atlanta, GA: CDC. http://www. cdc …; 2012.

3. World Health Organization. Global Tuberculosis Report. In: Annual Report. Geneva: WHO, 2020.

4. Connolly MiA, Gayer M, Ottmani S-E, Organization WH. Tuberculosis care and control in refugee and displaced populations: an interagency field manual: Geneva: World Health Organization; 2007.

5. Jackson IC. The 1951 Convention Relating to the Status of Refugees: A universal basis for protection. Int'l J Refugee L. 1991;3:403.

6. Urquia ML, Gagnon AJ. Glossary: migration and health. Journal of Epidemiology & Community Health. 2011;65(5):467-72.

7. Pavli A, Maltezou H. Health problems of newly arrived migrants and refugees in Europe. Journal of travel medicine. 2017;24(4).

8. UNHCR. UNHCR’s annual Global Trends report. 2018.

9. UNHCR. East and Horn of Africa 2018 end year report. 2018.

10. Figueroa-Munoz JI, Ramon-Pardo P. Tuberculosis control in vulnerable groups. Bulletin of the World Health Organization. 2008;86:733-5.

11. Pareek M, Greenaway C, Noori T, Munoz J, Zenner D. The impact of migration on tuberculosis epidemiology and control in high-income countries: a review. BMC medicine. 2016;14(1):48.

12. Rendon A, Centis R, Zellweger J-P, Solovic I, Torres-Duque C, Cordeiro CR, et al. Migration, TB control and elimination: whom to screen and treat. Pulmonology. 2018;24(2):99-105.

13. Liberati A, Altman DG, Tetzlaff J, Mulrow C, Gøtzsche PC, Ioannidis JP, et al. The PRISMA statement for reporting systematic reviews and meta-analyses of studies that evaluate health care interventions: explanation and elaboration. PLoS medicine. 2009;6(7):e1000100.

14. Modesti PA, Reboldi G, Cappuccio FP, Agyemang C, Remuzzi G, Rapi S, et al. Panethnic differences in blood pressure in Europe: a systematic review and meta-analysis. PloS one. 2016;11(1):e0147601.

15. Collaboration C. Cochrane Consumers and Communication Review Group: data extraction template for Cochrane reviews. Internet)(nd) Available from: cccrg cochrane org/author-resources. 2011.

16. Lu C-W, Lee Y-H, Pan Y-H, Chang H-H, Wu Y-C, Sheng W-H, et al. Tuberculosis among migrant workers in Taiwan. Globalization and health. 2019;15(1):18.

17. Asadi L, Heffernan C, Menzies D, Long R. Effectiveness of Canada's tuberculosis surveillance strategy in identifying immigrants at risk of developing and transmitting tuberculosis: a population-based retrospective cohort study. The Lancet Public Health. 2017;2(10):e450-e7.

18. Ospina JE, Orcau À, Millet J-P, Ros M, Gil S, Caylà JA, et al. Epidemiology of tuberculosis in immigrants in a large city with large-scale immigration (1991-2013). PloS one. 2016;11(10):e0164736.

19. Aldridge RW, Zenner D, White PJ, Williamson EJ, Muzyamba MC, Dhavan P, et al. Tuberculosis in migrants moving from high-incidence to low-incidence countries: a population-

based cohort study of 519 955 migrants screened before entry to England, Wales, and Northern Ireland. The Lancet. 2016;388(10059):2510-8.

20. Dierberg KL, Dorjee K, Salvo F, Cronin WA, Boddy JB, Cirillo D, et al. Improved detection of tuberculosis and multidrug-resistant tuberculosis among Tibetan refugees, India. Emerging infectious diseases. 2016;22(3):463.

21. Liu Y, Posey DL, Cetron MS, Painter JA. Effect of a culture-based screening algorithm on tuberculosis incidence in immigrants and refugees bound for the United States. Annals of internal medicine. 2015;162(6):420.

22. van den Boogaard J, Slump E, Schimmel HJ, van der Hoek W, van den Hof S, de Vries G. High Incidence of Active Tuberculosis in Asylum Seekers from Eritrea and Somalia in the First 5 Years after Arrival in the Netherlands. Emerging Infectious Diseases. 2020;26(4):675.

23. Vanino E, Tadolini M, Attard L, Po C, Francia F, Giannini A, et al. Systematic tuberculosis screening in asylum seekers in Italy. Clinical Infectious Diseases. 2017;65(8):1407-9.

24. Meier V, Artelt T, Cierpiol S, Gossner J, Scheithauer S. Tuberculosis in newly arrived asylum seekers: A prospective 12 month surveillance study at Friedland, Germany. International journal of hygiene and environmental health. 2016;219(8):811-5.

25. Aldridge RW, Zenner D, White PJ, Muzyamba MC, Loutet M, Dhavan P, et al. Prevalence of and risk factors for active tuberculosis in migrants screened before entry to the UK: a population-based cross-sectional study. The Lancet Infectious Diseases. 2016;16(8):962-70.

26. Ismail MB, Rafei R, Dabboussi F, Hamze M. Tuberculosis, war, and refugees: spotlight on the Syrian humanitarian crisis. PLoS pathogens. 2018;14(6):e1007014.

27. Trends G. Forced Displacement in 2015. UNHCR, The UN Refugee Agency, Geneva, Switzerland. 2016.

28. Asadi L, Heffernan C, Menzies D, Long R. Effectiveness of Canada's tuberculosis surveillance strategy in identifying immigrants at risk of developing and transmitting tuberculosis: a population-based retrospective cohort study. The Lancet Public health. 2017;2(10):e450-e7.

29. World Health Organization. European Centre for Disease Prevention and Control. Tuberculosis Surveillance and monitoring in Europe. 2014.

Figure

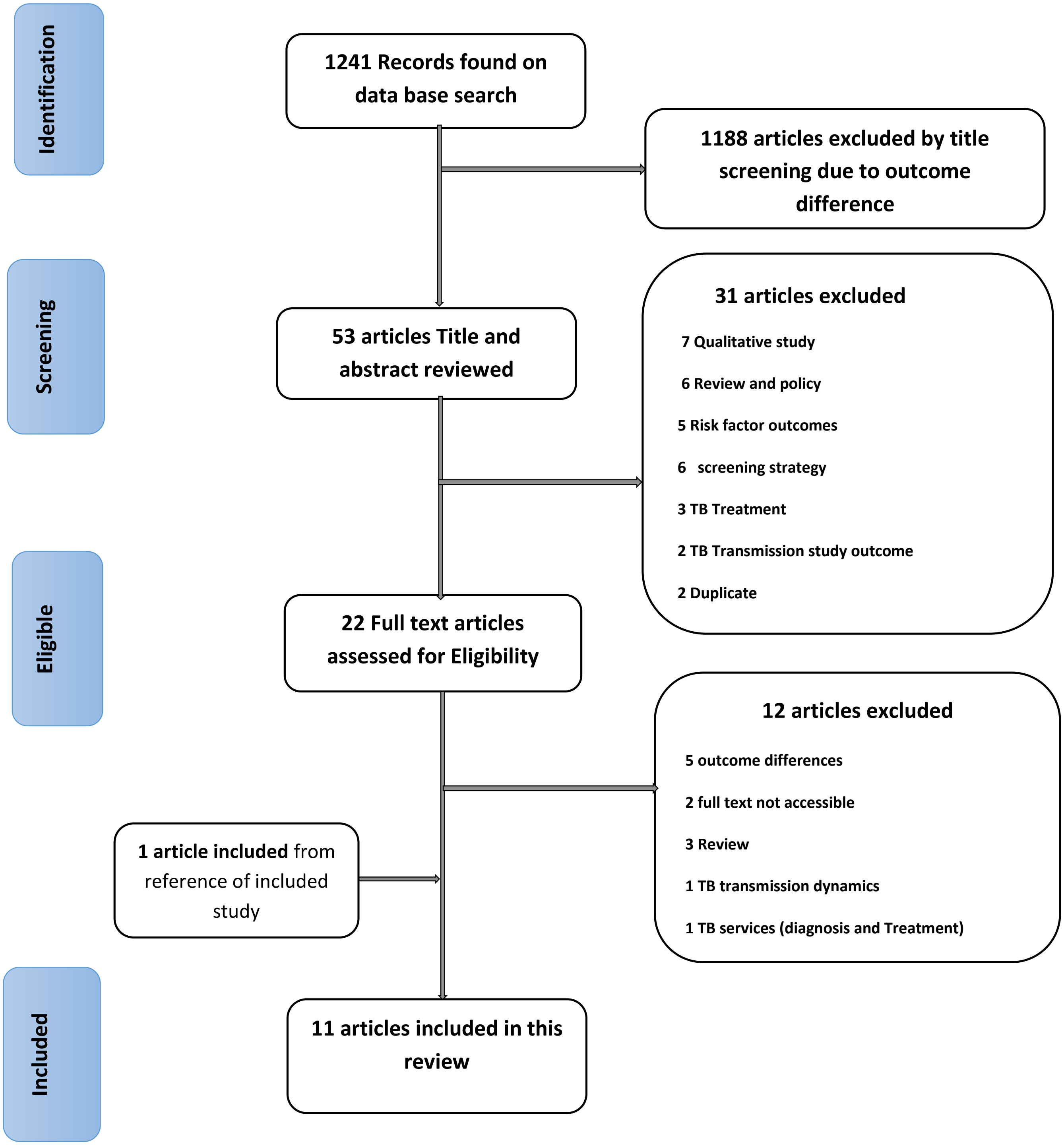

Figure 1. **Study selection process flow diagram**

Click here to access/download
Supporting Information
PRISMA_2020_checklist_Abyot Meaza.docx

Click here to access/download
Supporting Information
Study Quality assessment.docx

Click here to access/download
Supporting Information
PROSPERO_registered protocol_CRD42020157619.pdf
