# Supplementary material for: Tuberculosis among refugees and migrant populations: Systematic review
Source: PLoS One. 2022 Jun 9;17(6):e0268696. doi: 10.1371/journal.pone.0268696 (PMC9182295; doi:10.1371/journal.pone.0268696)
Supplement: Attachment — Submitted filename: Response to Reviewers.docx [file pone.0268696.s005.docx]

Date: April 01, 2022

From: Abyot Meaza (Author)

Subject: Submission of response to reviewers (PONE-D-21-33712).

I would like to thank the reviewers for the suggestions for correction and for comments for revision for the manuscript entitled “Tuberculosis among refugees and migrant populations: systematic review.”

**Response for Reviewer’s comment**

The authors presented systematic review aimed to summarize the evidence on the magnitude of TB among refugees and migrant populations. My comments were supplied in the PDF file of the manuscript. My decision is major revisions.

*We believe the comments help the quality of the manuscript. Therefore, all comments are accepted and submitted in revised versions (see Manuscript and Manuscript marked). Moreover, our response for the comments listed below in table.*

| Section | Comments | *Response* |
| --- | --- | --- |
| Abstract | Please rewrite the aim in brief | *Accepted and revised as indicated on page 2, in line 26-30* |
| Abstract | Why did the authors only talked the incidence in the abstract | *We presented incidence and prevalence as key findings and now we added additional finding as indicated on page 2 in line 38-39 and 41-44.* |
| Key words | Add more | *immigrants, asylum-seekers, and refugee-camps added as additional key words on page 3, line 51-52.* |
| Introduction | Add references to this sentence | *Four additional references (reference nr 13-16) added to support the stated statement as indicated on page 5, line 80.* |
| Methods, search strategy | Transfer to authors contribution | *Removed from page 6, line 105-106 and transferred to page 15, line 255-256.* |
| Methods, Study selection process | Transfer to authors contribution | *Removed from page 6, line 119-120 and transferred to page 15, line 256-257.* |
| Statistical Analysis | Please mention the program used | *Thank you for the comment and mentioned on page 8, 148-149.* |
| Results | Please provide the results with figures illustrating with results Analysis | *Accepted and provided as figure 2.* |
| Results | Please refer also to diagnosis and control of TB | *As per the included studies, the data supports to summarize the method of TB diagnosis and this has been summarized as indicated on page 9, 164-167 in addition to, in Table 1 and 2.* |
| Results | Tables not table | *Thank you for the comment and corrected on page 9, line 169 and page 11, line 178.* |
| Discussion | Explain | *The reason for the discussion point explained and supporting facts added on page 13, line 205-207 and reverence nr 33-35 cited* |
| Conclusion | Write a conclusion for the study | *The conclusion made as the end paragraph of discussion and now transferred as separate conclusion as indicated on page 15, line 246-51* |
| References | Add more recent references | *Accepted and 7 recent references added (reference cited from 13-16, and 33-35) as indicated on page 18 and page 20-21.* |

I appreciate and very grateful for your assistance.

Abyot Meaza

Researcher, Ethiopian Public Health Institute

PhD student, Addis Ababa University
